# Regional event-based surveillance in WHO’s Western Pacific Region

**DOI:** 10.5365/wpsar.2018.9.5.009

**Published:** 2020-06-30

**Authors:** Christopher Lowbridge, May Chiew, Katherine Russell, Takuya Yamagishi, Babatunde Olowokure, Li Ailan

**Affiliations:** a Division of Health Security and Emergencies, World Health Organization Regional Office for the Western Pacific, Manila, Philippines.

## Abstract

In the World Health Organization’s Western Pacific Region, event-based surveillance has been conducted for more than a decade to rapidly detect and assess public health events. This report describes the establishment and evolution of the Western Pacific Region’s event-based surveillance system and presents an analysis of public health events in the Region. Between July 2008 and June 2017, a total of 2396 events were reported in the Western Pacific Region, an average of 266 events per year. Infectious diseases in humans and animals accounted for the largest proportion of events recorded during this period (73%, 1743 events). Maintaining and strengthening this well established system is critical to support the rapid detection, assessment and response to public health events to sustain regional health security.

The early detection of public health events is critical to the implementation of rapid response measures to mitigate health, social and economic impacts. The effective detection and response to health emergencies is a key priority for the World Health Organization (WHO) and mandated to WHO under the International Health Regulations, IHR (2005). (*1*, *2*) The early detection of risks to public health is an important component of this, particularly in the context of today’s interconnected global community, in which even public health risks that originate in remote parts of the world may have an increased risk of spread. (*3*, *4*) No single country can undertake the task of regional surveillance and risk assessment. WHO, however, is well positioned to carry out this task. Public health surveillance is an essential component of WHO’s role in health emergencies, enabling the early detection, assessment and response to public health events, whether their impact is at the national, regional or global level. WHO works collaboratively with ministries of health, national public health agencies and other international organizations, for example, World Organization for Animal Health (OIE) and Food and Agriculture Organization of the United Nations (FAO).

Event-based surveillance (EBS) is the organized and rapid capture of information about events that are a potential risk to public health. (*5*) This information can be obtained through official or unofficial channels. Information from unofficial channels is usually unverified and non-standardized, being taken from sources such as media reports or community reporting. EBS reports require verification and then assessment before being used for public health purposes. Indicator-based surveillance is the consistent and systematic collection, monitoring, analysis and reporting of reliable data on diseases, syndromes and conditions from established, predominantly health-system-based formal sources, such as registers of notifiable diseases or syndromic surveillance systems. (*5*)

For more than a decade, the Asia Pacific Strategy for Emerging Diseases (APSED) (*6*) has guided Member States in the Western Pacific Region as a common framework for building the core capacities described in IHR (2005). (*2*) The Strategy includes a focus on regional preparedness, alerts and responses, which acknowledges and highlights the importance of both EBS and indicator-based surveillance to detect public health emergencies and gather information for risk assessment and public health decision-making. The Western Pacific Region’s surveillance system therefore uses multiple sources of information, both event-based and indicator-based, for risk assessment and decision-making for responses. (*6*)

While there have been various progress reports related to EBS as part of APSED implementation, existing WHO regional event detection, verification and risk assessment systems are not well described. This paper describes the Western Pacific Region’s surveillance and risk assessment system, in addition to presenting an analysis of events detected by the system between  July 2008 and June 2017.

## The evolution of event-based surveillance in the Western Pacific Region

### 2004–2005

In 2004, WHO’s Regional Office for the Western Pacific established a regional system for EBS, then known as rumour surveillance, following the first major emerging infectious disease outbreak of the 21st century: severe acute respiratory syndrome (known as SARS). This system was established with financial support from the Government of Japan to maintain one Field Epidemiology Training Programme (FETP) fellow to serve as a rumour surveillance officer, scanning media sources for rumours of potential public health risks daily. The major focus was infectious disease−related events.

### 2006–2015

The IHR were implemented to prevent, protect against, control and provide a public health response to the international spread of disease. (*2*) The IHR were revised in 2005, becoming the IHR (2005), and an obligation was added requiring State Parties to notify WHO of events that may constitute a public health emergency of international concern. IHR (2005) authorized WHO to seek verification from State Parties of unofficial reports of public health events. In addition, it established a network of national IHR focal points in Member States and IHR contact points within WHO to facilitate urgent reporting and communication about public health events. (*2*) The implementation of IHR (2005) led to a more systematic and formalized approach to rumour surveillance. (*2*) The Regional Office for the Western Pacific further strengthened event detection by building regional capacity, and it expanded its regional Field Epidemiology Fellowship Programme to include fellows and alumni of the FETP or the modified FETP (FET) from additional countries. The scope of event detection and assessments has also been expanded to cover more food safety and disaster events, including those caused by natural hazards, such as earthquakes and typhoons. In 2008, for the first time, the Regional Office published A Guide to Establishing Event-based Surveillance. (*5*)

### 2016 to the present

Lessons learnt from the 2014–2016 Ebola outbreak in West Africa led to the establishment of the WHO Health Emergencies (WHE) programme. The WHE programme provides a standard structure and mission across the Organization globally. The WHE programme includes a dedicated Health Emergency Information and Risk Assessment (HIM) unit for detecting events, assessing risks and managing information about emerging health threats. The WHE programme integrated the Regional Office for the Western Pacific’s EBS team into HIM and broadened the scope of event detection to include information management, using an all-hazards approach that includes outbreaks, emerging diseases, natural disasters, conflicts and other potential risks to human health.

## The Western Pacific Region’s event-based surveillance system

**Figure 1:**
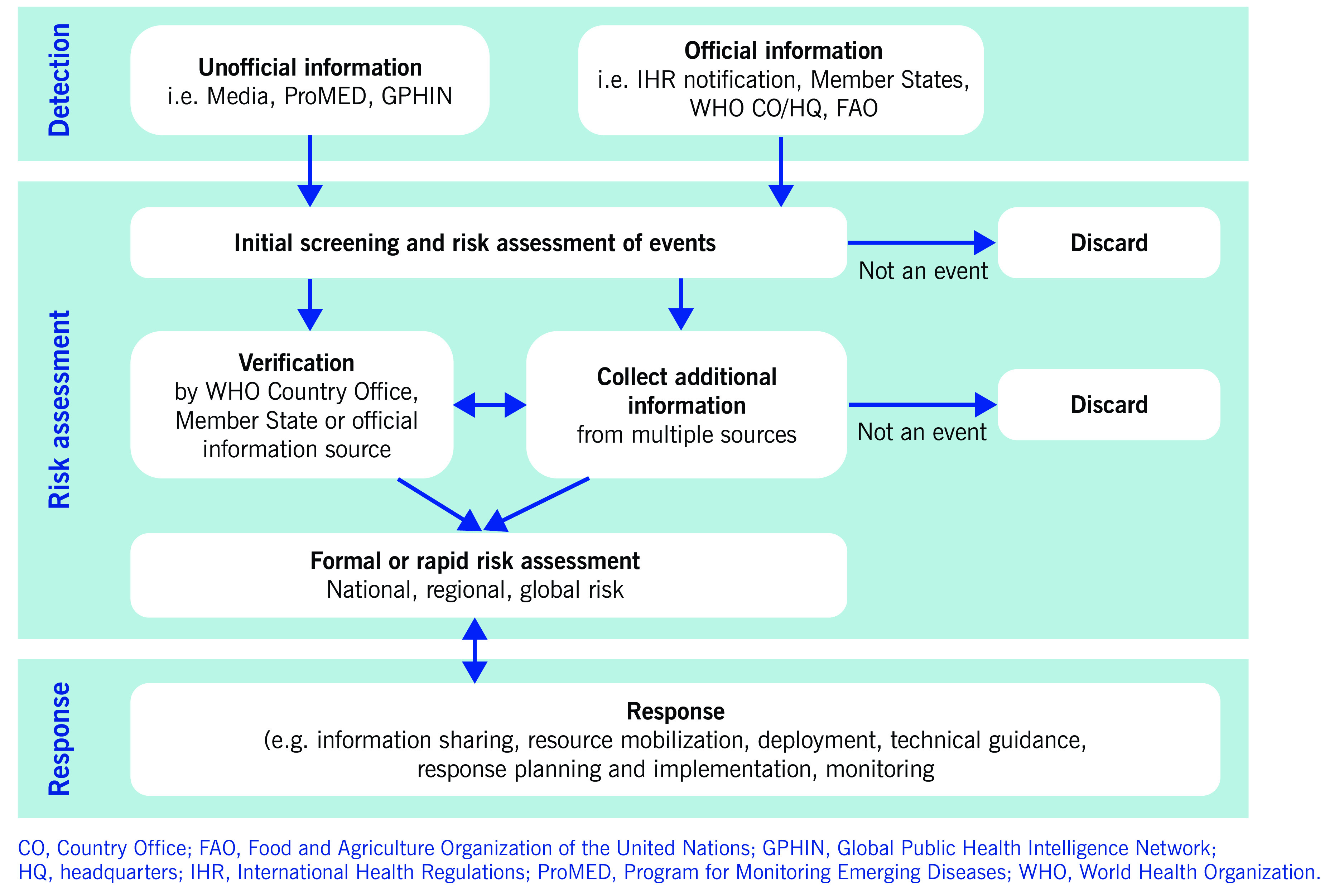
**WHO's Western Pacific Regional event-based surveillance, risk assessment and response system**

Since 2008, the Western Pacific’s regional EBS system has employed a standardized approach for surveillance, risk assessments and responses to public health events (**Fig. 1**). The system is operated by a team of epidemic intelligence officers, medical officers and epidemiologists. The epidemic intelligence officers include WHO staff and fellows from the regional FETP as well as professionals who have been seconded to the system, and volunteers and interns who have experience in communicable disease surveillance.

[**insert** Figure 1]

Event screening is undertaken twice daily, seven days a week. Information from both unofficial and official sources is screened using an event assessment tool (**Table 1**) that provides criteria for determining whether the information requires further assessment. Unofficial sources that are screened include internet-based early warning systems (e.g. the Global Public Health Intelligence Network, the Program for Monitoring Emerging Diseases [ProMED], and FluTrackers.com) and other web-based media sources. Official sources of information screened include communications from national IHR focal points to regional IHR contact points; WHO e-mail communications with country and regional offices, headquarters and collaborating centres; reports from partner agencies, such as international public health agencies and humanitarian and nongovernmental organizations; and surveillance reports, press releases and other official documents and reports from ministries of health that are shared with WHO or published online. To detect and monitor disasters and humanitarian emergency events, the Global Disaster Alerting Coordination System, Member States’ national disaster management offices, and web sites, such as ReliefWeb.int, are screened. Signals and events related to avian influenza are closely monitored within the Region. The web sites and media reports of the OIE and FAO are used to identify avian influenza events in animals within the Region and their potential public health risk.

**Table 1:** Regional event-based surveillance information screening tool used in WHO’s Western Pacific Region

1. Screening
• Screen all information sources for potential events daily

2. Assessment
Assess each piece of information against the following criteria. • Can the suspected disease cause outbreaks that have a high potential to spread (e.g. cholera, measles)? • Does the event involve a notifiable disease or defined notifiable syndrome with higher than expected morbidity or mortality? • Is the disease unusual or unexpected, or is there a new or unknown causal agent in the community? • Is there a cluster of cases or deaths with similar symptoms? • Could the event be caused by a product that is contaminated and commercially or widely available (e.g. a commercial food item)? • Does the event have possible consequences for trade or travel to or from the affected area? • Is there suspected spread of the infection in a health care or mass gathering setting? • If no human cases have been reported, does the event have a known or suspected consequence for human health (e.g. a chemical spill, unexplained deaths in animals)?

3. Outcome
• If the answers to all of the above criteria are no, then discard the information. • If the answer to one or more of the above criteria is yes or unknown, conduct additional assessments.

**Figure 2:**
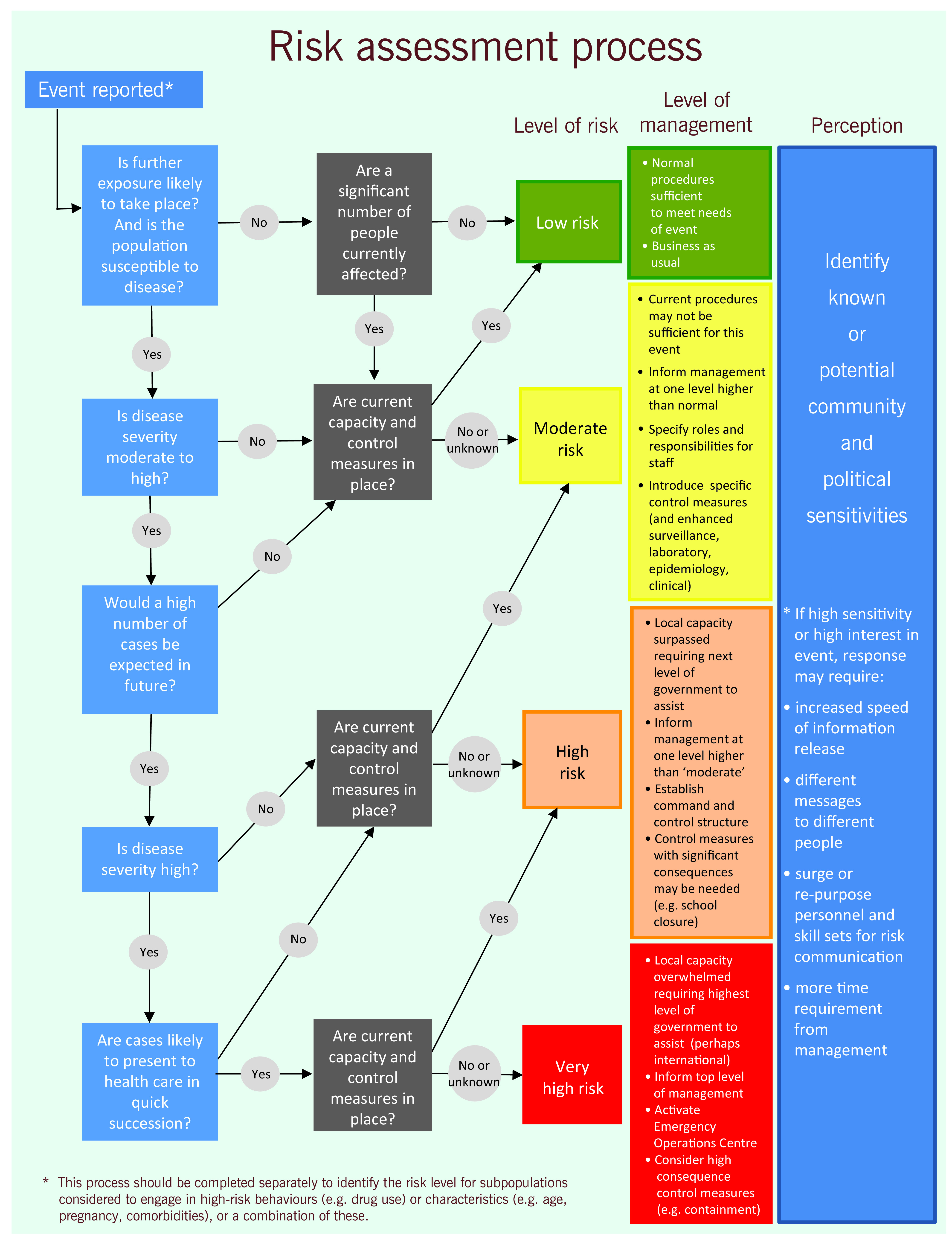
**WHO's Western Pacific Region algorithm for initial public health risk assessments**

**Figure 3:**
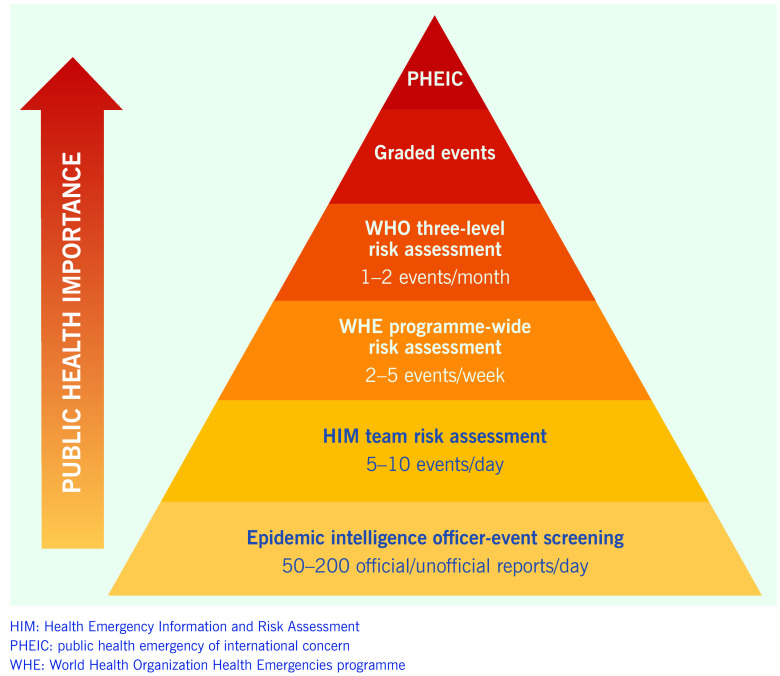
**The regional surveillance and risk assessment triangle used in WHO's Western Pacific Region**

Information that meets any two criteria within the event screening tool (**Table 1**) is assessed daily. This assessment includes using an algorithm-based risk assessment (**Fig. 2**) that determines whether an event may have implications for regional health security or there is a potential need for WHO support. Further assessment of the level of risk may be undertaken in relation to specific questions, as determined to be relevant to the event. Additional information may be obtained to inform the risk assessment, such as data on baseline disease incidence and contextual information about the setting in which the event is taking place. Events that are determined to pose a potential risk to public health are further reviewed by management and technical experts from within the WHE programme at the country, regional, subregional and global levels of WHO across the areas of epidemiology, laboratory expertise, risk communication, public health emergency preparedness, zoonoses, food safety, and emergency management, as well as by other technical divisions within WHO (**Fig. 3**).

[**insert** Figure 2]

[**insert** Figure 3]

In parallel with the initial internal WHO risk assessment process, verification of the information may be sought. Verification may involve confirming unofficial reports of an event with the national IHR focal point of a Member State or with the respective WHO country office. However, verification may also involve confirming an event through official information sources or through the triangulation of multiple unofficial or official information sources, or some combination of these.

The regional EBS system provides information and data with which to conduct the risk assessment, which is then used to make decisions about WHO’s response to public health events, in line with WHO’s emergency response framework. (*7*) Key response actions at the regional level may include conducting ongoing monitoring of the event; providing technical support; or deploying human, material or financial resources, or some combination of these, to affected countries and areas.

Events are entered into an internal EBS database daily. The EBS database serves as a repository of events with public health implications for the Western Pacific Region. Fields within the EBS database include event name, the class of hazard, disease, country affected, date of detection, and source of information. Daily, weekly and ad hoc summary and event-specific reports are produced by the HIM team and disseminated to all levels of WHO. The dissemination of these surveillance reports enhances situational awareness across WHO to improve readiness to respond to events when needed.

## Methods

A retrospective descriptive analysis of events in the EBS database in the Western Pacific Region was carried out for the period July 2008 through June 2017. This period was determined by the availability of data, and begins  1 year after the IHR (2005) came into force. In keeping with the Regional Office’s guidelines, events included clustered cases of a disease or syndromes, unusual patterns of disease or unexpected deaths, or situations that might lead to a potential exposure of humans to disease. (*5*) For the purposes of this report, events were classified into three categories: communicable diseases, avian influenza A(H5N1) outbreaks, and disasters and other events.

The number of new events by category was calculated for the study period by fiscal year (1 July to 31 June). A further analysis of events reported during the 2015 calendar year was conducted to determine the proportion that resulted in a response by Member States alone or with support from the WHO country office or Regional Office, or both. Between January and March 2016, data on the number of reports received by the surveillance system per day was collected to determine the average number of reports screened per day.

### Ethics statement

As this work is a report on routine EBS undertaken in line with IHR (2005) and does not involve human research, ethical clearance was not sought.

## Results

Between July 2008 and June 2017, a total of 2396 events were recorded in the EBS database (**Table 2**). Of these, 1176 (49%) were classified as infectious disease events, 653 (27%) were classified as disaster (all types) or other, and 567 (24%) were classified as avian influenza A(H5N1) events. An average of 266 events were recorded per year (range, 206 to 357 events). Between 2012 and 2017, the regional EBS system detected an average of 124 events related to influenza infection in either humans or animals. A selection of significant public health events detected by the surveillance system is listed in **Box 1**.

**Table 2:** Number (%) of events recorded in WHO’s Western Pacific Region event-based surveillance database, by year, 2008 to 2017^a^

Event type	Year																		Total	
	2008–2009		2009–2010		2010–2011		2011–2012		2012–2013		2013–2014		2014–2015		2015–2016		2016–2017			
Infectious diseases^b^	142	(69)	174	80%	206	(58)	114	(39)	47	(22)	67	(27)	70	(33)	208	(63)	148	(46)	1176	(49)
Avian influenza A(H5N1)	35	(17)	26	(12)	136	(38)	86	(29)	65	(31)	107	(43)	41	(19)	21	(6)	50	(16)	567	(24)
Disaster (all types)and other events^c^	29	(14)	18	(8)	15	(4)	94	(32)	99	(47)	72	(29)	101	(48)	102	(31)	123	(38)	653	(27)
**Total by year**	**206**	-	**218**	-	**357**	-	**294**	-	**211**	-	**246**	-	**212**	-	**331**	-	**321**	-	**2396**	-

^a^ A year is from 1 July to 30 June. The monitoring and reporting of disaster events became formalized in mid-2011. In 2013, they were further modified based on the   official criteria of the Centre for Research on the Epidemiology of Disasters (CRED) criteria.

^b^ Numbers exclude animal avian influenza events.

^c^ Category includes natural and other types of disasters. Other events included in this category include pharmaceutical related, food related, chemical and unknown or   unspecified.

Box 1 **Significant public health events detected by event-based surveillance in WHO’s Western Pacific Region, 2008−2017**

Implementation of the event-based surveillance system led to the early detection of, assessment of and response to several major health events, including:

- a large outbreak of enterovirus 71 in  Cambodia in 2012
- an outbreak of Middle East respiratory syndrome coronavirus in the Republic of Korea in 2015
- the spread of Zika virus disease within the Western Pacific Region in 2016
- a large outbreak of dengue in Solomon Islands in 2016
- an outbreak of measles in Papua New Guinea in 2017
- human infections with novel avian influenza viruses, including A(H7N9), in China.

**Figure 4:**
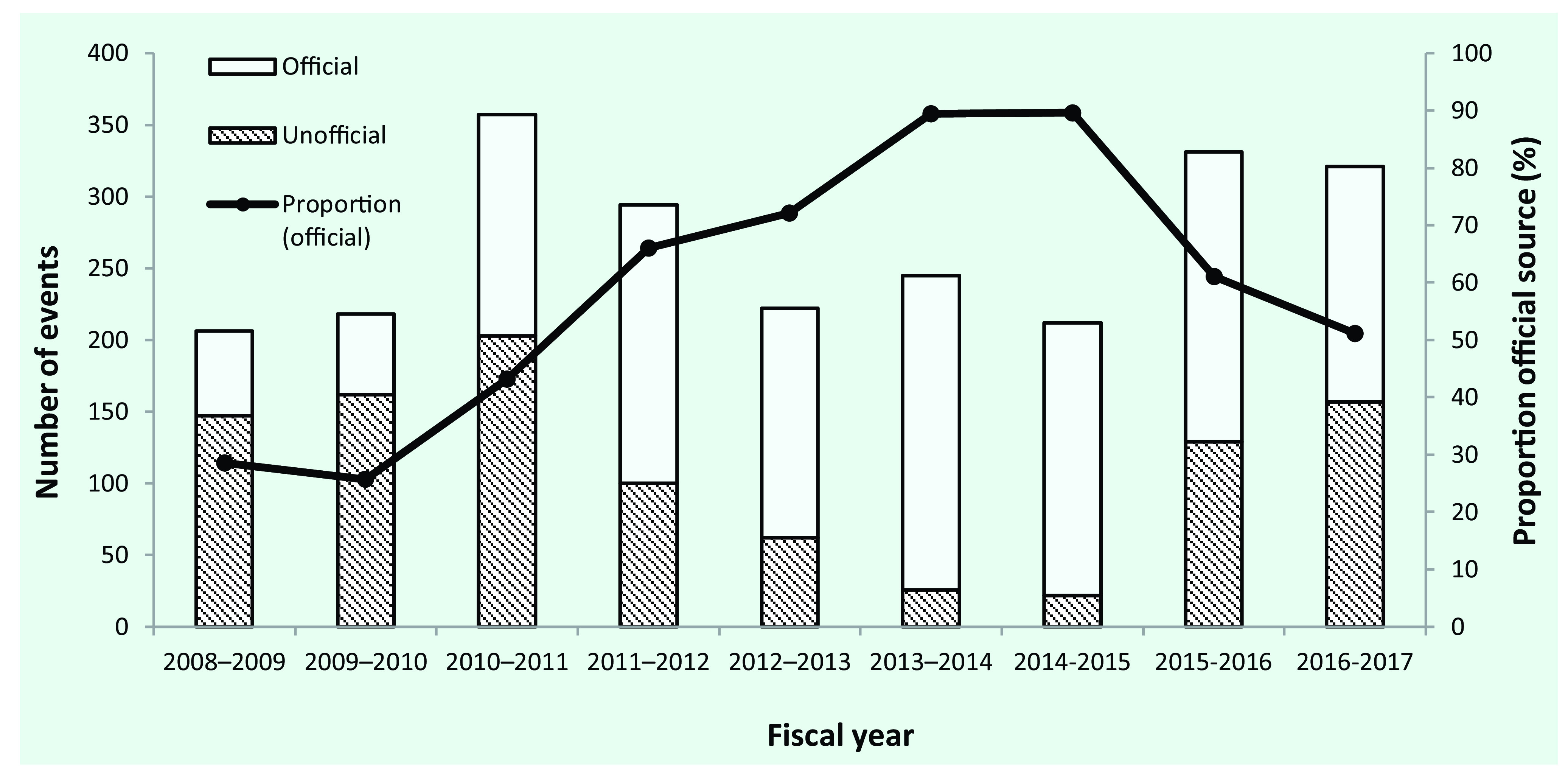
**Acute public health events in WHO's Western Pacific Region detected by official and unofficial information sources, by fiscal year, 2008–2017**

Between 2008 and 2017, 1398 (58%) events were detected from an official information source. There was an increasing trend in the proportion of events that were identified from official information sources up until 2014–2015, with a subsequent decline during 2015–2017 (**Fig. 4**).

[**insert** Figure 4]

In 2015, there were 218 public health events recorded in the database. Based on the records of these events, 131 (60%) were responded to by Member States without the support of WHO (although WHO monitored and assessed the events). Sixty-five (30%) were supported by WHO country offices, and 22 (10%) were supported either by WHO country, regional and headquarter offices or by the regional office if there was no country office.

## Discussion

Regional EBS and risk assessment are well established in WHO’s Regional Office for the Western Pacific, based on the substantial number of events that have been detected and responded to by WHO. It is a core function of the Regional Office to support event responses, including by providing technical support and deploying staff, material or financial resources. As such, EBS and risk assessment have been embedded within APSED. (*6*) Since the system’s beginnings as a basic rumour surveillance system, the Regional Office’s surveillance and risk assessment system has continuously evolved to detect signals earlier, assess risk more systematically, and manage information better. An analysis of the events reported to WHO under the IHR (2005) and published in WHO’s Disease Outbreak News reports, found a statistically significant improvement in the timeliness of outbreak discovery in the Western Pacific Region between 1996 and 2009.

The value of the regional EBS system’s ability to detect and assess information from multiple sources is highlighted by its applicability to influenza. For influenza, the regional EBS system captures events and signals from both the human and animal health sectors. Traditional and social media sources are monitored for early reports of severe acute respiratory infection or poultry die-off. Official reports from the OIE and the FAO are reviewed to detect and verify influenza events among animals. Reporting by Member States through the IHR (2005) mechanism is used to verify reports of human infection with avian influenza. The regional EBS system synthesizes information from these sources and others to provide timely and robust assessments and information to inform public health responses. In July and August 2017, the first poultry outbreaks of avian influenza A(H5N6) were detected in the Philippines. The regional EBS system synthesized information from the media, internal communications, OIE reports and official communications from the Philippines Department of Health to perform the risk assessment for this event.

Considerable effort has been made by WHO to strengthen the IHR (2005) core capacities of Member States within the Western Pacific Region through the implementation of APSED. An example that demonstrates the value of EBS and IHR (2005) reporting by Member States occurred in 2012 when a cluster of deaths among children of unknown etiology was notified through IHR (2005) by the Cambodian Ministry of Health. The etiology was later confirmed to be enterovirus 71. This event highlighted the benefit of the expanded scope of the IHR (2005) by using the IHR channel to report a public health event despite an unknown etiology. (*8*)

FETP and FET fellows and alumni in the Western Pacific Region have been crucial contributors to the regional surveillance system. Involving FETP and FET fellows and alumni from Member States in the Region as epidemic intelligence officers enables them to develop their skills and knowledge of EBS and risk assessment and also facilitates broader capacity-building in Member States through the dissemination of this knowledge within their respective countries.

Several limitations need to be considered when interpreting the results of our study. There is high turnover of staff within the surveillance system because FETP and FET fellows and alumni, volunteers and interns rotate every 2 months as part of the Regional Office’s on-the-job learning programme, and this may contribute to inconsistencies in data entry. Although there are standard operating procedures for epidemic intelligence officers, language, experience, technical knowledge and other factors may lead to differences in detection, accuracy and comprehensiveness. Furthermore, standard definitions and criteria for what constitute an event are lacking and vary depending on the hazard type. For disasters, the Centre for Research on the Epidemiology of Disasters (CRED) criteria (*9*) were used, and these criteria differed from those used for public health events related to infectious diseases. The CRED criteria may have made officers more sensitive to including disasters in the database. With the adoption of an all-hazards approach within the WHE programme, there have been increasing efforts to monitor small-scale disasters in the Region, which may account for the increasing trend seen in such events within the database. The number of animal outbreaks is an underestimate because during the earlier years of data collection, only avian influenza A(H5N1) events were recorded.

APSED III, a revision of APSED (2015), was published in 2017 and aims to further strengthen surveillance to support Member States in the Western Pacific Region. (*6*) The availability of new and innovative technologies for data management offers opportunities to improve surveillance systems, both through streamlining current processes for data management and providing enhanced functionality for analysis and reporting. To ensure that the regional surveillance system meets the needs of Member States, partners and internal stakeholders within WHO, particularly, those in country offices, we recommend ongoing evaluation and monitoring of the system.

## Conclusions

This 10-year analysis of the Western Pacific Region’s EBS system illustrates its functions in early detection and risk assessment of all-hazard public health events by using information from diverse official and unofficial sources. Maintaining this well established surveillance system is critical to support rapid detection, assessment and responses to public health events, thus maintaining and advancing health security in WHO’s Western Pacific Region and globally. As such, the Regional Office for the Western Pacific continues to strengthen its function as the hub for regional surveillance and risk assessment to better serve the needs of Member States.
